# Bitter taste receptors in the gut-vascular axis: a novel target for immune and metabolic regulation of hypertension

**DOI:** 10.3389/fimmu.2025.1714761

**Published:** 2026-01-13

**Authors:** Xiaoyu Wei, Feifei Lyu, Fuyun Jia, Lihong Zhang, Min Zhang, Qiang Xu, Shengyu Hua

**Affiliations:** 1 College of Traditional Chinese Medicine, Tianjin University of Traditional Chinese Medicine, Tianjin, China; 2 Department of Traditional Chinese Medicine, The Sixth Medical Center of PLA General Hospital, Beijing, China; 3 Department of cardiology, Second Affiliated Hospital of Tianjin University of Traditional Chinese Medicine, Tianjin, China; 4 Department of geratology, Affiliated Hospital of Tianjin Academy of Traditional Chinese Medicine, Tianjin, China

**Keywords:** gut-vascular axis, hypertension, immunometabolism, macrophage polarization, Tas2Rs, therapeutic targets

## Abstract

Hypertension affects approximately 1.3 billion adults worldwide, yet the control rate remains below 20%, highlighting the limitation of current therapies that primarily lower blood pressure without targeting the underlying pathophysiological mechanisms. Recent research indicates that bitter taste receptors, which are structurally distinct members of the G protein-coupled receptor superfamily, have functions that extend beyond their traditional role in oral taste perception. These receptors are extensively expressed along the gut-vascular axis, including in vascular smooth muscle, cardiac tissue, macrophages, and gastrointestinal organs, thereby positioning them as crucial nodes in the regulation of immunometabolic processes. This review systematically elucidates the complex regulatory mechanisms of gut-vascular axis TAS2Rs in the pathophysiology of hypertension and investigates TAS2R-targeting compounds, with particular emphasis on their effects in modulating blood pressure. This review consolidates evidence on TAS2R signaling across vascular, immune, and gastrointestinal interfaces to outline therapeutic implications for hypertension.

## 1 Introduction

Hypertension, a leading modifiable risk factor for global premature mortality, affects an estimated 1.3 billion adults worldwide, representing nearly 30% of the adult population and contributing significantly to cardiovascular diseases (1–4). Despite available treatments, its prevalence has doubled since 1990, with awareness and control rates remaining near 50% and 20%, respectively. It was responsible for approximately 11 million deaths in 2019, accounting for 20% of global mortality (5, 6). Defined by elevated systolic and/or diastolic blood pressure, hypertension is primarily idiopathic (90% of cases), with pathogenesis involving a complex interplay of genetic, environmental, and neurohormonal factors (7–10). It is increasingly conceptualized as a multifactorial syndrome arising from dysregulated neuroendocrine-immune interactions (11). Although current pharmacotherapy includes six main drug classes and can reduce blood pressure and target organ damage, approximately half of patients fail to achieve adequate control to mitigate cardiovascular risk (12, 13). Importantly, current treatments primarily aim at lowering blood pressure rather than targeting the underlying pathophysiological mechanisms of hypertension (14), resulting in decreased long-term adherence to medication (15), adverse effects related to treatment, and persistent uncontrolled hypertension (16, 17). Consequently, there is a pressing need to identify novel therapeutic targets that address both the symptoms and the underlying pathophysiological mechanisms concurrently.

In recent years, as our understanding of the complex pathophysiological mechanisms underlying hypertension has deepened, the concept of the gut-vascular axis has emerged as a prominent area of research. Initially introduced by Flori L, this concept describes a dynamic, bidirectional regulatory network that encompasses both physiological and pathological interactions between the gut and vascular systems (18). The gut-vascular axis can be defined as a sophisticated communication system facilitated by various signaling molecules, including microbial metabolites, hormones, neurotransmitters, and immune factors. These molecules establish connections between the gut microbiota, intestinal endocrine cells, intestinal immune cells, and the intestinal barrier system with systemic vascular endothelium, smooth muscle, myocardium, and immune cells. The primary function of this axis is the coordinated regulation of vascular tone, inflammatory responses, and blood pressure homeostasis (18–20). This axis not only underscores the intricate relationship between the gut and the vasculature but also elucidates how imbalances in the gut microenvironment can drive pathological processes in cardiovascular diseases, such as hypertension, through multiple pathways.

The gut-vascular axis plays a significant role in the development and progression of hypertension. Gut microbiota dysbiosis exacerbates hypertension through the gut-vascular axis via two interlinked mechanisms. Firstly, microbial imbalance compromises intestinal barrier function, leading to the systemic translocation of substances such as lipopolysaccharide (LPS), which in turn triggers systemic inflammation and sympathetic nervous system activation, ultimately impairing vascular function (18, 21, 22). Secondly, the microbiota influences the secretion of gastrointestinal hormones (e.g., Glucagon-Like Peptide-1, GLP-1), and dysbiosis weakens their associated vasodilatory and anti-inflammatory effects (23–28). These pathways intertwine, forming a vicious cycle that promotes hypertension. In conclusion, the gut-vascular axis operates as an integrative physiological network that seamlessly connects the gut microbiome, metabolites, immune responses, hormonal signaling, and vascular components, collectively influencing the maintenance or disruption of blood pressure homeostasis.

**Figure 1:**
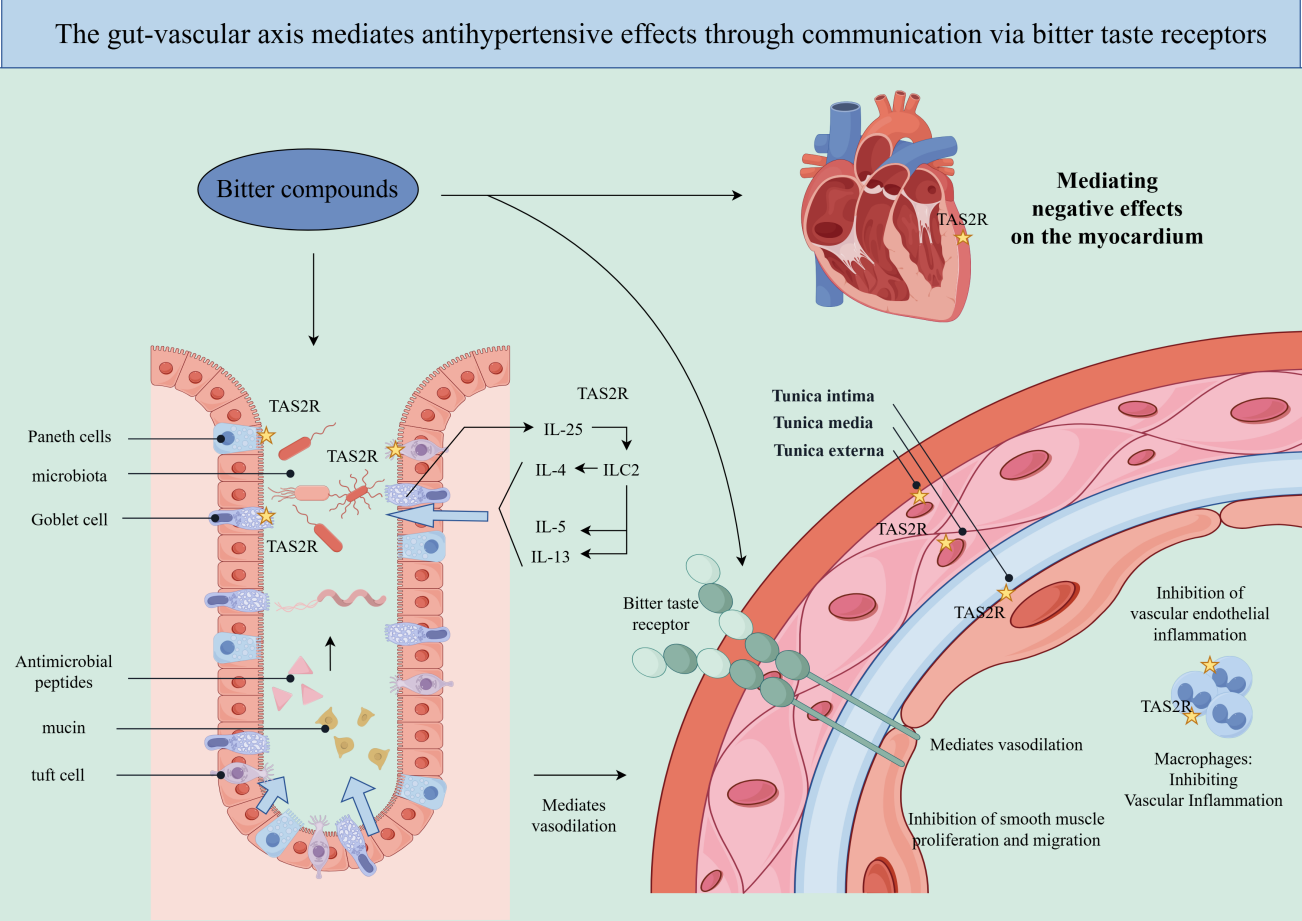
Gut-vascular communication via TAS2R: Bitter taste receptors are expressed at multiple interfaces of the gut-vascular axis, and modulating these receptors can achieve a synergistic blood pressure-lowering effect.

Recent research has demonstrated that TAS2Rs, which are structurally distinct members of the G protein-coupled receptor (GPCR) superfamily, are extensively expressed beyond the oral cavity, including in arterial vessels and the gastrointestinal tract. These receptors extend beyond their traditional role in taste perception and have been shown to play pivotal roles in regulating vascular dilation, metabolic homeostasis, and immunomodulatory pathways (29). Notably, the direct regulation of vascular tone by TAS2Rs indicates a significant and mechanistically substantiated link to the pathophysiology of hypertension (30–32). This finding necessitates a reassessment of the therapeutic potential of these multifunctional receptors in the treatment of hypertension. Bitter taste receptors exhibit multifaceted roles in the regulation of hypertension. At the vascular-cardiac interface, these receptors located on vascular smooth muscle cells facilitate vasodilation (30, 31), inhibit the proliferation of smooth muscle cells, and prevent vascular remodeling (33, 34). Additionally, bitter taste receptors on macrophages contribute to immunomodulation and the suppression of vascular inflammation (35, 36). In cardiac tissue, they are involved in mediating negative inotropic effects and reducing heart rate (37, 38). At the gastrointestinal interface, bitter taste receptors play a crucial role in maintaining gut microbiota homeostasis and promoting the secretion of hormones that lower blood pressure (39–41). Due to their diverse regulatory functions across multiple interfaces, bitter taste receptors are emerging as highly promising therapeutic targets within the gut-vascular axis for the management of hypertension (**Figure 1**).

Given the intricate nature of hypertension pathogenesis, addressing all its facets may be impractical. Consequently, this review adopts an external perspective to introduce a novel paradigm in the study of bitter taste receptors and hypertension. It reconceptualizes these receptors as crucial regulators within the gut-vascular axis and delineates essential translational pathways from bitter taste receptor-mediated immunometabolic regulatory mechanisms to clinical applications. The review investigates the following critical questions: (1) How do bitter taste receptors within the gut-vascular axis lower blood pressure via immunometabolic mechanisms? (2) What is the translational potential of activating bitter taste receptors? (3) What unmet clinical needs and future directions exist for clinical translation?

## 2 Bitter taste receptor overview

Humans possess the ability to perceive sweet, umami, bitter, salty, and sour tastes. Notably, the perception of bitter taste functions as one of the most fundamental chemical defense mechanisms in the course of biological evolution, significantly contributing to the avoidance of toxin ingestion by eliciting aversive responses upon the consumption of potentially harmful substances (42, 43). The primary mediators of this process, known as bitter taste receptors, have extended beyond their conventional gustatory roles and are now recognized as crucial molecular switches that play a significant role in the regulation of various physiological systems (44).

**Figure 2:**
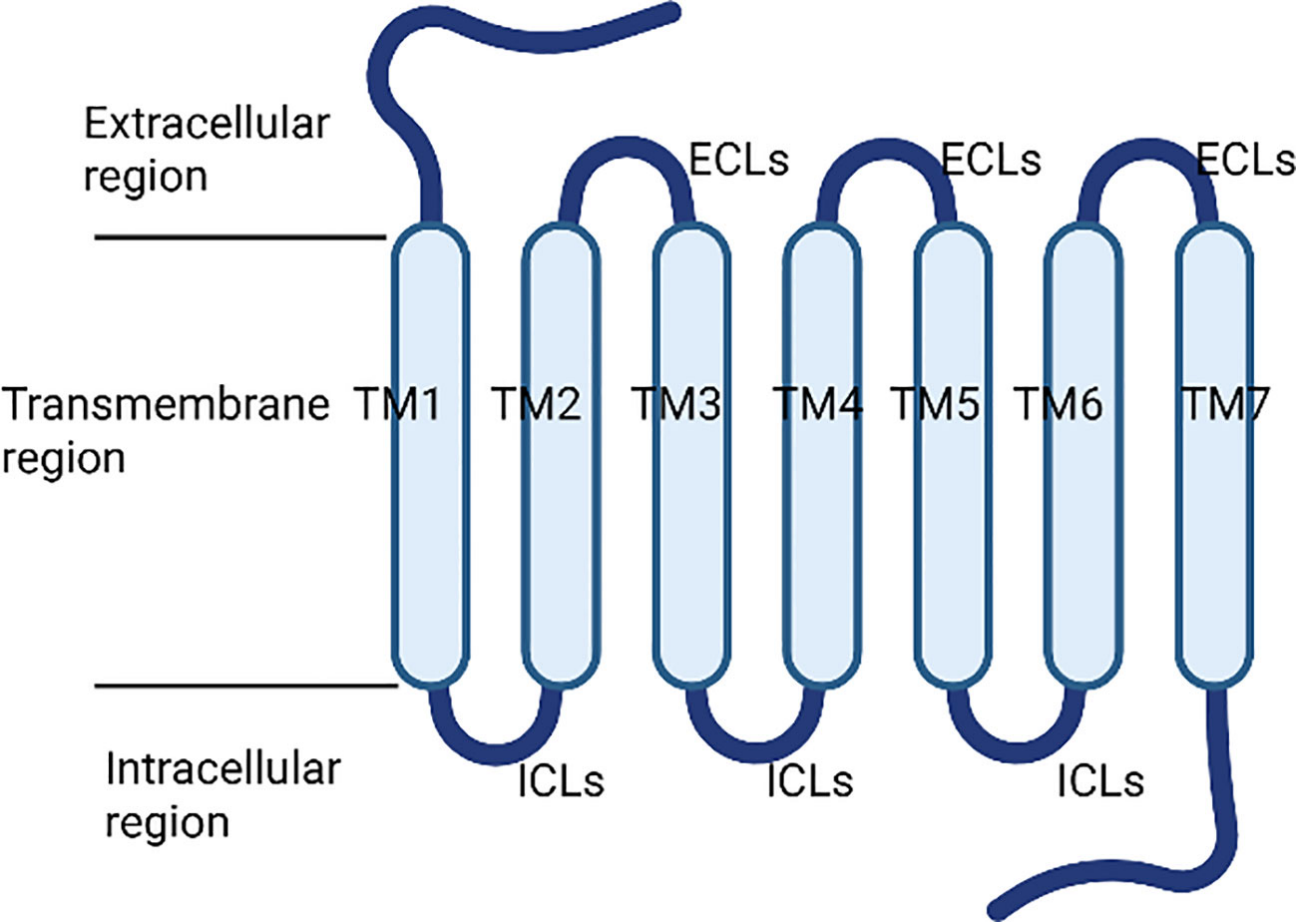
Structures of bitter taste receptors.

Bitter taste receptors, also known as TAS2Rs, represent a specialized subfamily within the GPCR superfamily, specifically classified under the Class T subfamily (45). Initially identified in porcine tongue tissue in 1969 (46), these receptors were formally acknowledged as members of the GPCR family in 2000 (47, 48). The fundamental structure of TAS2Rs comprises a single polypeptide chain that is organized into seven transmembrane helical domains (TMs). These domains are interconnected by three extracellular loops (ECLs) and three intracellular loops (ICLs), which link the short extracellular N-terminus to the intracellular C-terminus (49, 50). The transmembrane domains and extracellular loops collectively form the ligand-binding domain, characterized by significant polymorphism (**Figure 2**). In contrast, the intracellular loops are highly conserved and play a crucial role in coupling with taste-specific G proteins, such as α-gustducin, to initiate downstream signaling pathways (51, 52).

The human genome encodes 25 functional TAS2R subtypes, which are organized into clusters on chromosomes 5, 7, and 12 (53–55). The amino acid sequence similarity among these subtypes varies between 30% and 70% (56), with significant diversity observed in the ECL regions. In contrast, the transmembrane domains (e.g., TM1, TM3, TM7) and the intracellular loop 2 (ICL2) contain conserved motifs, such as LxxxR in TM2 and LxxSL in TM5 (57–59). This structural variation facilitates subtype-specific ligand recognition profiles, which are categorized based on their agonist response range: broad-spectrum receptors (e.g., TAS2R14/38/46) respond to more than 16 compounds, intermediate-spectrum receptors (e.g., TAS2R39/40/43) respond to 6–16 compounds, narrow-spectrum receptors (e.g., TAS2R3/5/8) respond to 1–3 compounds, and orphan receptors (e.g., TAS2R42/45/48) have no known ligands (60, 61).

The primary physiological role of TAS2R is to detect potentially toxic substances, such as alkaloids, phenols, and lactones, thereby establishing a defensive mechanism through aversive reactions (43). Research indicates that individual TAS2R receptors can identify multiple structurally diverse bitter compounds (**Table 1**); for instance, hTAS2R4 is responsive to 33 different compounds. Conversely, individual compounds, such as quinine, can activate multiple receptor subtypes (44, 51). Nevertheless, no single compound has been identified that activates all TAS2R subtypes, nor are there agonists that selectively activate a single specific TAS2R subtype. Importantly, TAS2Rs, as structurally distinct entities within the GPCR superfamily, are expressed beyond the gustatory system and are widely distributed across various systemic tissues and cells. In these locations, they mediate novel physiological and pathological processes, including vasodilation, metabolic regulation, and immune responses (29).

**Table 1:** Agonists of bitter taste receptors.

Bitter taste receptor subtype	GeneCards	Receptor agonist
T2R1	*TAS2R1*	Diphenidol, Thiamine, Chloramphenicol, Lupulone, Quinine, Arborescin, Parthenolide, Amarogentin, etc
T2R2	*TAS2R2*	Chlorhexidine, Picrotoxinin, Propylthiouracil, Phenylbutazone, Yohimbine hydrochloride, etc
T2R3	*TAS2R3*	Chloroquine, Soyasaponin I
T2R4	*TAS2R4*	Quinine, Azathioprine, Chlorpheniramine, Brucine, Artemorin, Quassin, Colchicine, etc
T2R5	*TAS2R5*	Epigallocatechin gallate, (-)-Epicatechin, tannins, Punicalagin, 1,10-phenanthroline, etc
T2R7	*TAS2R7*	Magnesium Sulfate, Quinine, Caffeine, Papaverine, Diphenhydramine, Strychnine, Dextromethorphan, Chloroquine, Quercetin, etc
T2R8	*TAS2R8*	Denatonium benzoate, Chloramphenicol, Andrographolide, Oleuropein, Ligstroside aglycon, Ritonavir, etc
T2R9	*TAS2R9*	Ofloxacin, Procainamid, Pirenzapin, etc
T2R10	*TAS2R10*	Quinine, Benzoin, Absinthin, Arglabin, Caffeine, Coumarin, Cucurbitacin B, Parthenolide, Quassin, Diphenidol, etc
T2R13	*TAS2R13*	Denatonium benzoate, Diphenidol, Lopinavir, Oxyphenonium, etc
T2R14	*TAS2R14*	Lupulone, Humulone, Benzoin, Quinine, Absinthin, Arborescin, Aristolochic acid, Caffeine, Noscapine, Diphenidol, Picrotoxinin, etc
T2R16	*TAS2R16*	Diphenidol, Sodium Benzoate, Amygdalin, Arbutin, Sinigrin, Gentiobiose, etc
T2R38	*TAS2R38*	Diphenidol, Chlorpheniramine, Berberine, Yohimbine, Limonin, Sodium thiocyanate, Acetylthiourea, allyl isothiocyanate, Methimazole, etc
T2R39	*TAS2R39*	Tenofovir alafenamide, Resveratrol, Herbacetin, Cyanidin chloride, Acetylgenistin, Vanillin, Theaflavin, Ranitidine, Taurocholic acid, Pyrocatechin, Sulfuretin, etc
T2R40	*TAS2R40*	Quinine, Aristolochic acid, Diphenidol, Cohumulone, Isoxanthohumol, Fisetin, Quercetin, etc
T2R41	*TAS2R41*	Quinine, Diphenidol, Denatonium, Wogonin, Aristolochic acid, Parthenolide, etc
T2R42	*TAS2R42*	Soyasaponin I
T2R43	*TAS2R43*	Quinine, Arborescin, Aristolochic acid, Caffeine, Diphenidol, Denatonium benzoate, Amarogentin, Aloin, Quercetin, Apigenin, Fisetin, Lactucin, etc
T2R44	*TAS2R44*	Quinine, Aristolochic acid, Diphenidol, Andrographolide, Quercetin, Fisetin, etc
T2R45	*TAS2R45*	-
T2R46	*TAS2R46*	Quinine, Denatonium benzoate, Absinthin, Colchicine, Arborescin, Caffeine, Diphenidol, Picrotoxinin, Brucine, Strychnine, Cnicin, etc
T2R47	*TAS2R47*	Amarogentin, Denatonium benzoate, Protocatechuic acid, Diphenidol, Cohumulone, Picrotoxinin, Brucine, etc
T2R48	*TAS2R48*	-
T2R49	*TAS2R49*	Diphenidol, Xanthotoxin, Vanillin, etc
T2R50	*TAS2R50*	Dehydroandrographolide, Amarogentin, Andrographolide, etc
T2R60	*TAS2R60*	-

Refer to BitterDB (http://bitterdb.agri.huji.ac.il).

## 3 Distribution of bitter taste receptors in the gut-vascular axis

TAS2Rs are predominantly localized in the plasma membrane of Type II taste receptor cells (TRCs) within the taste buds located on the tongue, soft palate, and pharynx (62–64). The binding of bitter compounds to these receptors initiates transduction cascades that convey signals to the brain, ultimately resulting in the perception of bitter taste (65). Notably, TAS2Rs are also expressed along the human gut-vascular axis, including in arteries (31, 66, 67), the heart (38, 68–70), macrophages (58, 67, 71), and the gastrointestinal tract (72–75). At these various sites, TAS2Rs fulfill diverse roles in modulating immunity and metabolism, as well as in the regulation of blood pressure (**Table 2**).

**Table 2:** Distribution of bitter taste receptors in the gut - vascular axis.

Gut - vascular axis	Localization	TAS2R	Reference
Vascular interface	Heart	TAS2R10, TAS2R14, TAS2R30, TAS2R31, TAS2R46, TAS2R50	(38, 68–70)
	Arteries	TAS2R1, TAS2R3, TAS2R4, TAS2R5, TAS2R7, TAS2R8, TAS2R9, TAS2R10, TAS2R13, TAS2R14, TAS2R39, TAS2R40, TAS2R42, TAS2R50, TAS2R60	(31, 66, 67)
	Macrophages	TAS2R1, TAS2R3, TAS2R4, TAS2R5, TAS2R7, TAS2R8, TAS2R9, TAS2R13, TAS2R16, TAS2R19, TAS2R30, TAS2R31, TAS2R38, TAS2R42, TAS2R44, TAS2R45	(58, 67, 71)
Gastrointestinal interface	Stomach	TAS2R1, TAS2R3, TAS2R4, TAS2R5, TAS2R7, TAS2R9, TAS2R10, TAS2R13, TAS2R14, TAS2R16, TAS2R30, TAS2R31, TAS2R38, TAS2R39, TAS2R40, TAS2R41, TAS2R42, TAS2R43, TAS2R46, TAS2R50	(72–75)
	Duodenum	TAS2R1, TAS2R3, TAS2R4, TAS2R5, TAS2R14, TAS2R19, TAS2R20, TAS2R38, TAS2R46	(72–75)
	Jejunum	TAS2R14, TAS2R38, TAS2R43	(72–75)
	Ileum	TAS2R1, TAS2R3, TAS2R4, TAS2R5, TAS2R19, TAS2R20, TAS2R38, TAS2R46	(72–75)
	Colon	TAS2R1, TAS2R3, TAS2R4, TAS2R5, TAS2R19, TAS2R20, TAS2R38, TAS2R46	(72–75)

## 4 Regulatory mechanisms and therapeutic opportunities of bitter taste receptors in the gut-vascular axis for hypertension

### 4.1 Bitter taste receptors in the vascular (cardiac) interface

Recent studies have confirmed that TAS2Rs, a subset of G protein-coupled receptors, are extensively distributed within the cardiovascular system and play a crucial role in mediating significant antihypertensive effects through various mechanisms. Specifically, agonists of TAS2Rs have been shown to induce relaxation of vascular smooth muscle, inhibit vascular remodeling, exert negative inotropic effects on the heart, and modulate immune-inflammatory responses. The multi-target regulatory capabilities of TAS2Rs offer innovative strategies for the treatment of hypertension, addressing the limitations associated with conventional pharmacological interventions.

#### 4.1.1 Bitter taste receptor-mediated vascular smooth muscle relaxation

Recent research has advanced our understanding of the functional roles of TAS2Rs within the vascular system. Traditionally thought to be confined to oral tissues for the perception of bitter taste, numerous studies have now demonstrated that TAS2Rs are extensively distributed in extra-oral tissues, including the vasculature. TAS2R subtypes are ubiquitously expressed in vascular endothelium and smooth muscle tissues, as confirmed across various species. Evidence indicates that TAS2R agonists significantly induce dilation of vascular smooth muscle (76). Importantly, the vasodilatory mechanisms vary among specific agonists: chloroquine and noscapine exert their effects through a unique caveolae-dependent signaling pathway, while denatonium and quinine primarily induce vasodilation by antagonizing α-adrenergic receptors—an action independent of endothelial function, BKCa channel activation, or L-type calcium channel blockade. Notably, conflicting evidence suggests quinine’s effect may partially depend on endothelium-mediated signaling pathways. Additionally, flufenamic acid-induced vasodilation involves nitric oxide (NO) signaling and potassium channel activation (30). Accumulating evidence indicates that TAS2R agonists, as novel vascular relaxants, exert a core effect by inducing vasodilation. This has been confirmed across various models: from rat and guinea pig aortas to pulmonary arteries, agonists such as chloroquine and denatonium benzoate can induce potent relaxation of vascular smooth muscle and reduce blood pressure *in vivo* (31, 77, 78). Clinically, reduced bitter taste sensitivity in hypertensive patients is associated with long-term high salt intake, while enhancing bitter taste signaling through pathways such as TRPM5 may help ameliorate cardiovascular dysfunction and hypertension induced by high salt intake (79, 80). Additionally, the benefits of specific TAS2R subtype agonists extend beyond mere blood pressure reduction. For example, agonists of TAS2R10 and TAS2R38 have also been shown to protect target organs such as the heart and kidneys (81, 82). More interestingly, the expression of TAS2R10 in penile blood vessels and the potent relaxing effects of its agonists offer the potential for dual benefits in treating erectile dysfunction (ED), which is common in hypertensive patients (83, 84). Although relaxing vascular smooth muscle is important, regulating the vascular endothelium is equally interesting (85); however, there is currently a lack of evidence for TAS2R regulating endothelial cell function. Current research limitations remain: The mechanism of TAS2R agonist-induced vasodilation primarily involves calcium channel modulation, overlapping with the mechanism of clinical calcium channel blockers. While studies confirm chloroquine blocks voltage-dependent L-type Ca^2+^ channels, its potency and specificity differ from conventional calcium antagonists. Future research must clearly distinguish between specific receptor-mediated effects of TAS2R agonists and nonspecific calcium channel blockade. Moreover, elucidating the subtype-specific mechanisms of TAS2Rs in different vascular beds, developing higher-selectivity agonists, and evaluating their clinical potential for hypertension treatment are urgently needed.

#### 4.1.2 Bitter taste receptor inhibition of vascular smooth muscle proliferation and migration

Hypertension is frequently associated with pathological alterations in arterial structure and function, notably characterized by arterial wall stiffening and endothelial dysfunction. These changes adversely affect hemodynamics, leading to impaired blood perfusion to end-organs and consequently elevating the risk of disease and mortality in patients (86). A bidirectional pathological relationship exists between hypertension and increased arterial stiffness/vascular remodeling; these vascular structural and functional abnormalities can act both as initiating factors for hypertension and as consequences of its progression, thereby creating a mutually reinforcing vicious cycle (87, 88). Although the causal relationship between arterial stiffening, vascular remodeling, and hypertension remains a subject of debate (89), the early inhibition of vascular remodeling is crucial, irrespective of causality (90). It is well recognized that vascular smooth muscle cells (VSMCs) are the primary cellular constituents of arteries and exhibit plasticity, with VSMCs proliferation being fundamental to hypertension-associated vascular remodeling (6, 91).

**Figure 3:**
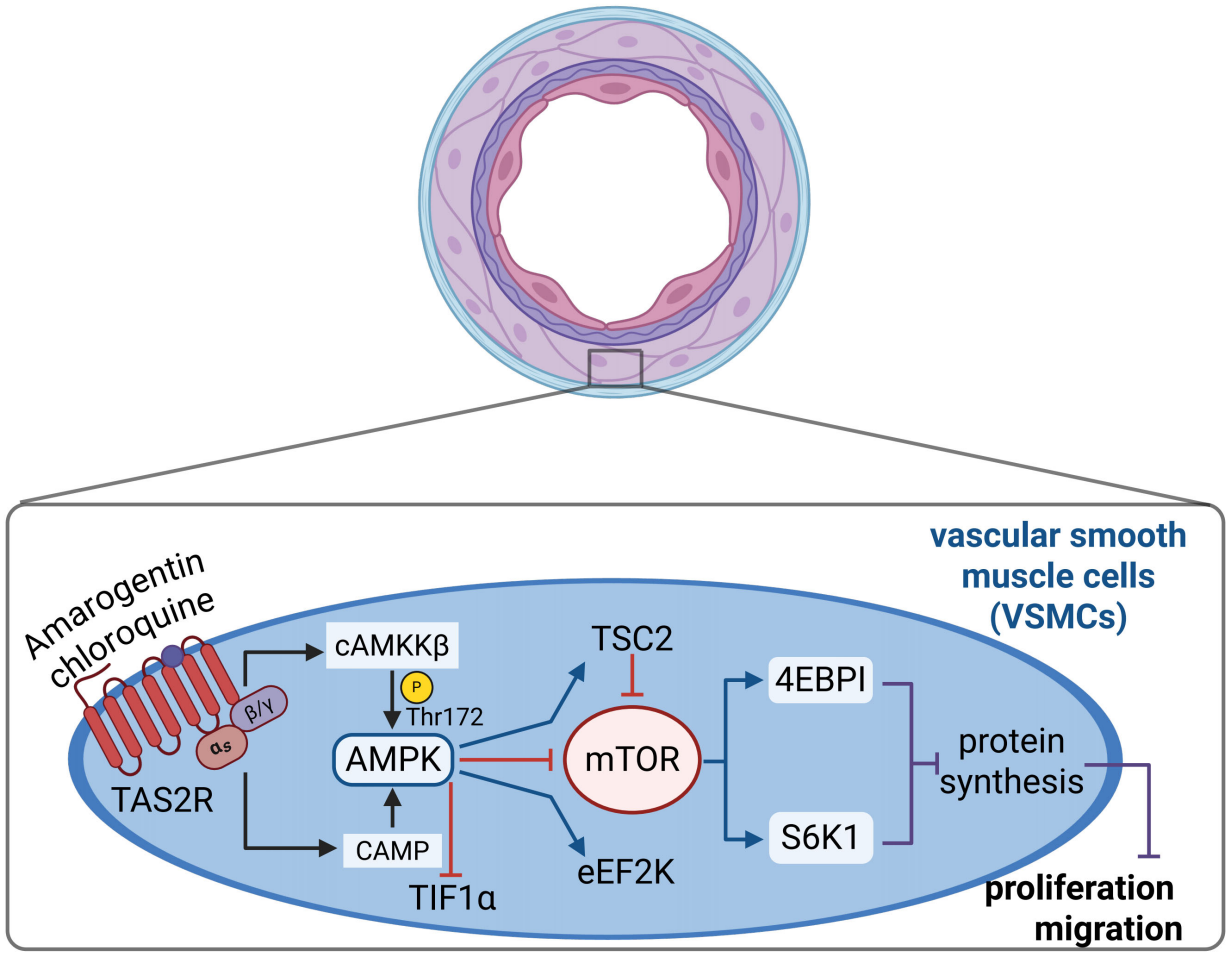
Potential mechanisms of TAS2R inhibiting vascular smooth muscle proliferation and migration: Amarogentin and chloroquine activate the AMPK signaling pathway in VSMCs through TAS2R, after which phosphorylated AMPK regulates mTOR, thereby inhibiting the proliferation and migration of VSMCs (103, 104). mTOR: rapamycin complex. Figures created with BioRender.com.

Emerging evidence substantiates the critical role of TAS2R activation in the inhibition of cell proliferation and the facilitation of apoptosis (33, 34). TAS2Rs are widely expressed in various cancer cells such as colorectal and breast cancer (34, 92–95). Their activation can induce apoptosis through mechanisms like triggering nuclear Ca^2+^ responses, mitochondrial depolarization, and caspase activation, demonstrating significant potential for cancer therapy (96–100). This anti-proliferative effect also applies to smooth muscle cells. In airway smooth muscle, TAS2R activation promotes apoptosis by suppressing cell cycle gene expression and downregulating pathways such as pERK1/2 (101, 102). More importantly, in vascular smooth muscle, agonists like amarogentin and chloroquine induce apoptosis via the AMPK pathway activation (**Figure 3**), thereby effectively inhibiting the proliferation and migration of vascular smooth muscle cells (103, 104). This provides a highly promising therapeutic strategy for intervening in hypertension-associated vascular remodeling. Nonetheless, current research is largely limited to *in vitro* cell models and rodent studies, leaving the applicability of these findings to human hypertension pathophysiology uncertain.

#### 4.1.3 Bitter taste receptor-mediated cardiac negative inotropic effects

**Figure 4:**
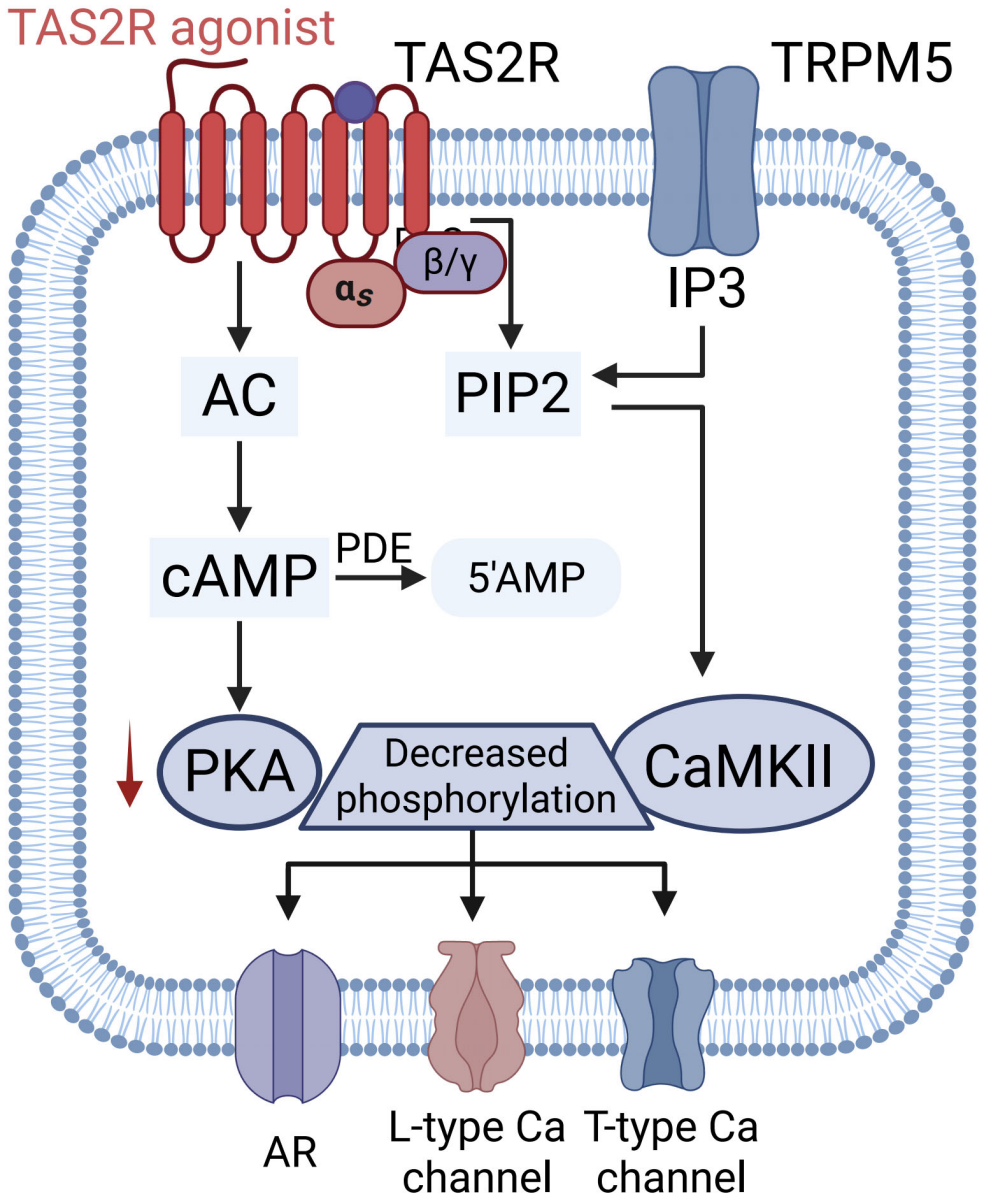
Potential mechanisms of bitter taste receptor-mediated cardiac negative inotropic effect: Activation of TAS2R leads to the dissociation of Gα-gustducin and Gβγ; Gα-gustducin activates membrane adenylyl cyclase (AC) to produce cAMP, but also enhances cAMP hydrolysis via a phosphodiesterase (PDE)-dependent pathway, thereby reducing protein kinase A (PKA) expression and diminishing its activation of Ca^2+^/calmodulin-dependent protein kinase II (CaMKII). This cascade ultimately results in the downregulation of ion channels and receptors critical for heart rate regulation, including L-type Ca^2+^ channels (LTCCs), TRPM5, and adrenoceptors (AR), leading to a deceleration of sinoatrial node cell rhythm and the inhibition of tachycardia.

Sinus tachycardia (resting heart rate > 100 bpm) is an independent risk factor for hypertension and cardiovascular diseases (105–107). Currently, beta-blockers and calcium channel blockers are first-line medications for simultaneously controlling heart rate and blood pressure (108). The activation of TAS2Rs offers a highly promising new strategy in this regard. Studies have shown that TAS2R agonists exert cardiovascular protective effects through two pathways. First, they produce a negative inotropic effect on the heart, reducing cardiac workload and blood pressure by decreasing stroke volume and cardiac output (37, 38). Second, TAS2Rs can directly modulate cardiac electrical activity. Agonists act through a complex signaling cascade, ultimately downregulating the expression of key ion channels and receptors such as L-type Ca^2+^ channels, TRPM5, and adrenergic receptors, thereby slowing sinus node rhythm and effectively suppressing tachycardia (**Figure 4**) (109, 110). This dual action of concurrently lowering heart rate and contractility produces pharmacological effects similar to those of beta-blockers and calcium channel blockers, positioning TAS2Rs as a potential novel therapeutic target for essential hypertension, hypertension complicated by tachycardia, and related heart failure. Nonetheless, current research is constrained by significant limitations: TAS2R exhibit relatively low mRNA expression levels in cardiac tissue (111), which not only complicates the reliable detection of their protein expression but also obstructs comprehensive functional investigation. Additionally, the functional roles of TAS2R within the heart remain inadequately characterized, underscoring the necessity for future studies to integrate protein expression validation with functional analysis.

#### 4.1.4 Bitter taste receptor inhibition of vascular inflammation

##### 4.1.4.1 Bitter taste receptors on vascular endothelium regulate immune inflammation

**Figure 5:**
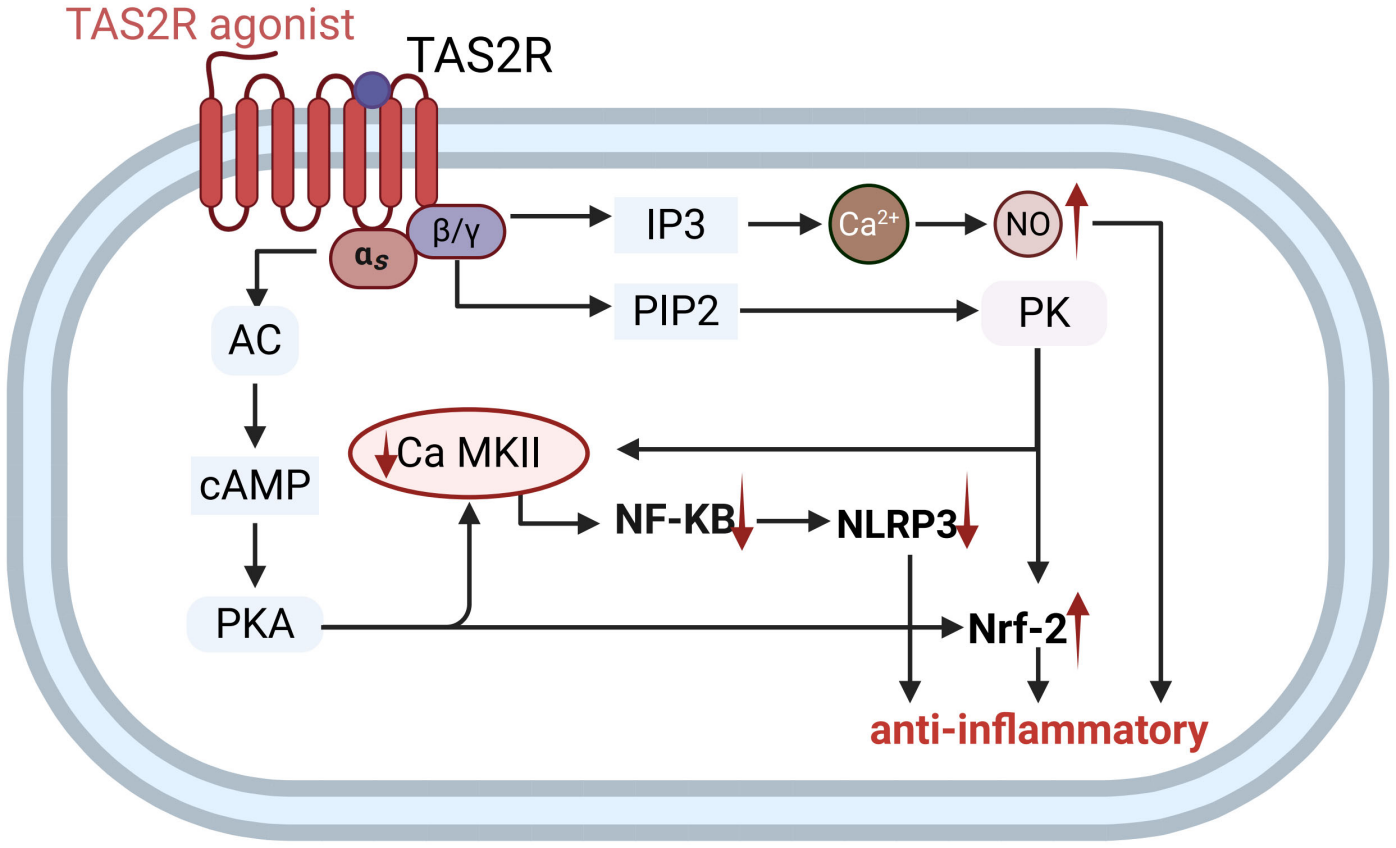
Potential mechanisms of bitter taste receptors on vascular endothelium in regulating immune inflammation: Agonist-induced activation of TAS2Rs initiates dual signaling cascades that converge to suppress cardiovascular inflammation. Upon agonist binding, TAS2Rs couple to Gα-gustducin, stimulating adenylate cyclase (AC) to elevate cyclic AMP (cAMP) levels and activate protein kinase A (PKA). Concurrently, TAS2Rs release Gβγ subunits, which activate phospholipase Cβ (PLCβ). PLCβ hydrolyzes phosphatidylinositol 4,5-bisphosphate (PIP_2_) to generate inositol trisphosphate (IP_3_) and diacylglycerol (DAG), triggering intracellular Ca^2+^ release and protein kinase (PK) activation. These parallel pathways, Gα-gustducin/AC/cAMP/PKA and Gβγ/PLCβ/PIP_2_/PK, orchestrate two critical anti-inflammatory mechanisms. First, they induce downregulation of Ca^2+^/calmodulin-dependent kinase II (CaMKII) expression, leading to inhibition of nuclear factor-kappa B (NF-κB) activity and subsequent suppression of NLRP3 inflammasome transcription, thereby attenuating pro-inflammatory cytokine release. Second, they facilitate nuclear factor erythroid 2-related factor 2 (Nrf-2) nuclear translocation, enhancing antioxidant response element (ARE) driven gene expression to further suppress inflammation.

A growing body of research evidence suggests that the pathogenic mechanisms underlying essential hypertension are multifactorial. Notably, the activation of the immune system has been identified as a significant contributor to the elevation of blood pressure, primarily through the induction of vascular inflammatory responses and microvascular remodeling. This process has emerged as a crucial component in the onset and progression of hypertension (112). Vascular endothelial cells (VECs), as critical interfaces in immune responses, can detect pathogen and damage signals and initiate inflammatory reactions (112–114). TAS2Rs expressed on VECs enable them to sense various metabolites, such as bacterial quorum-sensing molecules (QSMs) and advanced glycation end products, thereby playing a role in regulating cardiovascular inflammation (115, 116). During cardiovascular inflammation, nuclear factor-κB (NF-κB) and NOD-like receptor pyrin domain-containing protein 3 (NLRP3) inflammasome activation drive pro-inflammatory responses, whereas the oxidative stress sensor nuclear factor erythroid 2-related factor 2 (Nrf2) counteracts this process by upregulating antioxidant and anti-inflammatory mediators through cross-regulation with these pathways (117–119). In the context of vascular inflammation, it has been documented that the expression of TAS2Rs and key downstream signaling molecules is reduced (32, 120). The activation of TAS2Rs exerts potent anti-inflammatory effects through a dual mechanism (**Figure 5**): on one hand, it inhibits the key NF-κB/NLRP3 inflammatory axis via known signaling pathways, thereby reducing the production of inflammatory factors; on the other hand, it promotes the nuclear translocation of the antioxidant factor Nrf2, enhancing cellular antioxidant and anti-inflammatory capacities (110, 121, 122). Additionally, TAS2R agonists may synergistically inhibit inflammation by activating eNOS to produce nitric oxide (NO), although this mechanism has not yet been confirmed in vascular systems (123). Although this mechanism has been confirmed only in the context of respiratory epithelial cell inflammation, we hypothesize that it may similarly apply to vascular endothelial cells. It is important to note that current research on TAS2R immunoregulatory mechanisms is predominantly focused on the respiratory system. While studies suggest that vascular endothelial cells are involved in innate immunity and express TAS2R (55), there is a lack of direct evidence elucidating the specific mechanisms of endothelial TAS2R in hypertension. Consequently, future research is urgently needed to explore the pathophysiological associations and to elucidate the underlying mechanisms.

##### 4.1.4.2 Bitter taste receptors on macrophages regulate immune inflammation

The causal relationship between immune cells and hypertension has been widely confirmed, with immune cells serving as key regulators in the vascular endothelial microenvironment, mediating and sustaining elevated blood pressure (124–126). Similar to endothelial cells, immune cells recognize pathogen- and damage-associated molecular patterns via core pattern recognition receptors to initiate responses (127, 128), and they also express multiple TAS2R subtypes, further enhancing their defensive functions (72). Hypertension is a chronic low-grade inflammatory disease in which macrophages play a central role (129). As the primary defense line of innate immunity, macrophages exhibit remarkable plasticity, polarizing into pro-inflammatory M1 phenotypes or anti-inflammatory/tissue-repair M2 phenotypes (130, 131). In hypertension, macrophages contribute to the disease through various mechanisms, such as M-CSF, ROS, RAAS, and salt sensitivity (132), thus, regulating their polarization is a crucial therapeutic strategy (133). This is confirmed by the significant activation of M1 macrophages in various hypertensive animal models and the subsequent restoration of blood pressure through pharmacological inhibition of this activation (134), a finding also preliminarily supported by clinical data (135).

**Figure 6:**
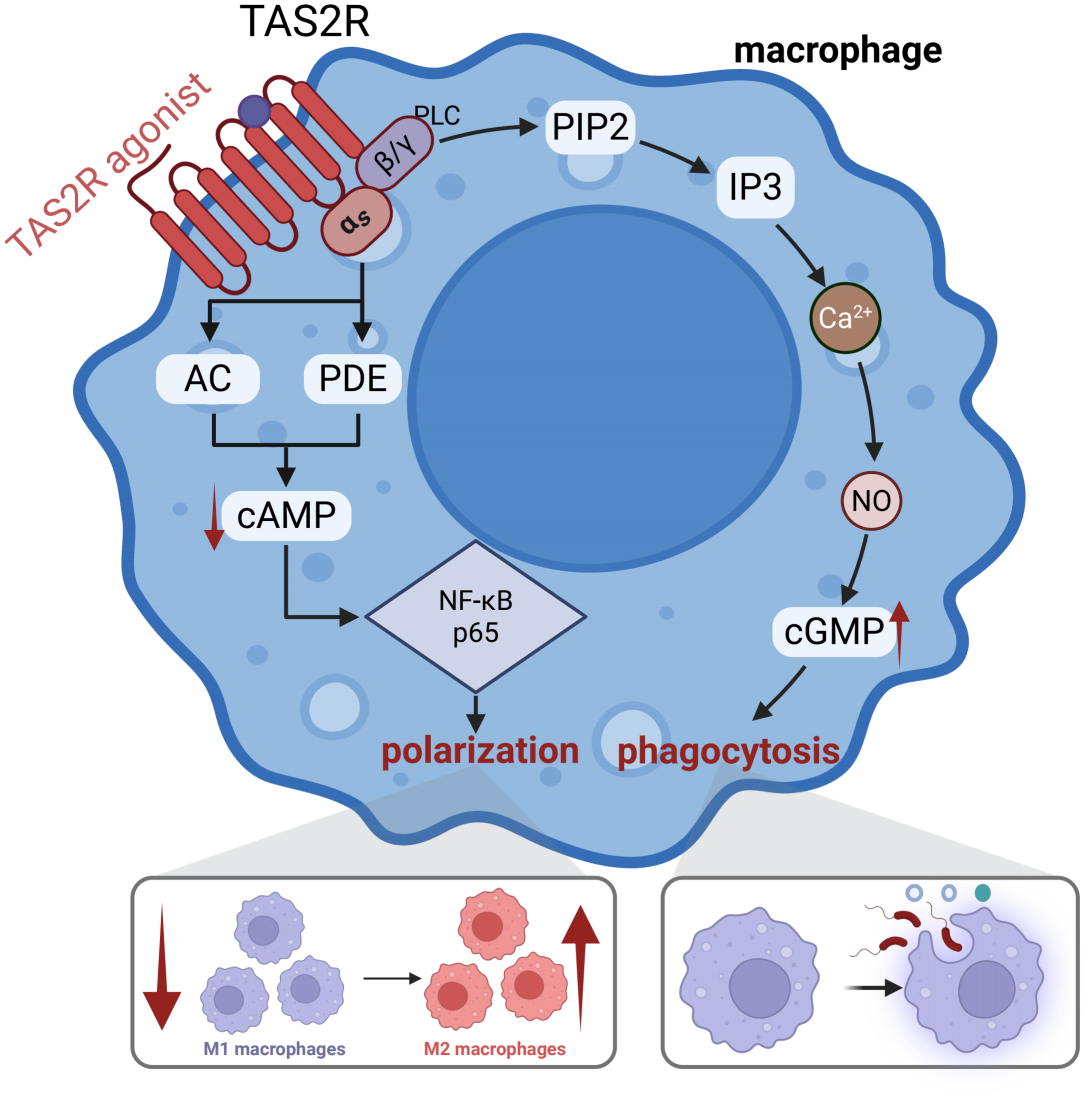
Potential mechanisms of TAS2R on Macrophages Regulating Immune Inflammation: After TAS2Rs on the macrophage surface are activated, Gβγ stimulates a cascade increasing Ca^2+^, which activates nitric oxide synthase (NOS) to produce NO, elevating cGMP. Concurrently, the Gαi subunit (or related Gα subunits like Gαgust) reduces cAMP levels, either by directly inhibiting adenylyl cyclase (AC) via Gαi or by activating phosphodiesterase (PDE) via Gαgust. These combined changes ultimately enhance macrophage phagocytosis. Meanwhile, the decrease in cAMP can inhibit LPS-induced phosphorylation of NF-κB p65, thereby modulating macrophage polarization (136, 137).

Macrophages are key targets for TAS2R modulation. Sixteen TAS2R subtypes have been identified in macrophages, with their expression upregulated upon stimulation (72). The application of TAS2R agonists can significantly inhibit macrophage infiltration, enhance phagocytic activity, suppress the release of M1-associated factors (such as TNF-α, CCL3, and CXCL8), and promote the expression of M2-associated factors, thereby contributing to blood pressure reduction (**Figure 6**) (35, 36). This effect is primarily mediated by NO and cAMP signaling pathways (136, 137). Moreover, TAS2Rs can protect monocyte/macrophage DNA from oxidative stress damage and promote the differentiation of monocytes into M2 macrophages (138). For instance, polyphenols from Kuding tea have been shown to promote M2 differentiation and exert antihypertensive effects via TAS2Rs (139). In summary, TAS2Rs are widely expressed in macrophages and are essential for their phagocytic activity and phenotypic polarization (136, 137), indicating significant therapeutic potential in managing hypertension-associated inflammation. Nevertheless, the variability in TAS2R expression and their intricate mechanisms across different stages of hypertension and among diverse hypertensive populations remains insufficiently understood, thereby constraining their translational potential. Additionally, when stimulated by various agonists and within distinct microenvironments, TAS2Rs may demonstrate pathway preferences that differentially modulate macrophage functions. Consequently, further empirical research is required to substantiate the modulation of macrophage phagocytosis and polarization via TAS2R activation in the context of chronic inflammation associated with hypertension.

### 4.2 bidirectional gut-vascular signaling modulated by TAS2Rs

TAS2R located at the gastrointestinal interface are pivotal in mediating antihypertensive effects by modulating the composition of gut microbiota and enhancing the secretion of gastrointestinal hormones. Activation of TAS2R orchestrates the equilibrium of bacterial, fungal, and viral communities within the gut, suppresses the colonization of pathobionts, and fosters the proliferation of beneficial bacteria, thereby impacting pathways that regulate blood pressure. Concurrently, this receptor signaling pathway effectively induces the release of hormones such as GLP-1, ghrelin, and cholecystokinin, which collectively contribute to blood pressure reduction through mechanisms including vasodilation, sympathetic inhibition, and metabolic regulation. The TAS2R-mediated gut-vascular axis thus offers a significant theoretical framework and translational potential for targeted interventions in the management of hypertension.

#### 4.2.1 Bitter taste receptor regulation of gut microbiota

The gut microbiota encompasses a diverse array of microorganisms, including bacteria, archaea, fungi, and viruses (140), and plays a pivotal role in human health. Recent studies have identified associations between gut microbiota and hypertension, with dysbiosis of the gut microbiota being considered a potential etiological factor in the development of hypertension (141, 142). The majority of the gut microbiota is made up of gut bacteria. Research indicates that a higher presence of bacterial genera like *Lactobacillus*, *Roseburia*, *Coprococcus*, *Akkermansia*, and *Bifidobacterium* is linked to lower blood pressure, while higher levels of *Streptococcus*, *Blautia*, and *Prevotella* are strongly linked to higher blood pressure (143). Traditional research has predominantly concentrated on the gut bacterial community, leaving the roles of the gut mycobiota and gut virome in blood pressure regulation relatively unexplored. Despite comprising a minor fraction of the total gut microbiota, recent studies have increasingly highlighted their significant biological functions in maintaining blood pressure homeostasis (144, 145). Importantly, the gut virome demonstrates heightened sensitivity compared to the bacterial community in the early detection of hypertension (146). For example, the abundance of specific viruses, such as *Mimivirus* and *Deltaentomopoxvirus*, shows a positive correlation with systolic blood pressure (SBP), whereas the *Betterkatz virus* is significantly linked to increased diastolic blood pressure (DBP) (147). Concurrently, an increasing body of evidence underscores the relationship between gut mycobiota and hypertension. Research indicates that certain gut fungi can induce metabolic disorders and may directly or indirectly contribute to elevated blood pressure. Although no statistically significant differences in fungal alpha diversity have been identified between hypertensive patients and healthy controls (148, 149), alterations in the abundance of specific fungal taxa are strongly associated with blood pressure levels. For instance, genera such as *Malassezia*, *Exophiala*, *Mesospora*, and *Saccharomyces* have been implicated in pathophysiological associations with increased blood pressure. Conversely, the *genus Mortierella* may confer cardiovascular protective effects by modulating lipid metabolism or anti-inflammatory pathways (148–150). Nonetheless, the study of the relationships between the gut virome/mycobiota and hypertension is still in its exploratory stages, necessitating further research to determine causality. Overall, a growing body of evidence indicates that dysbiotic gut microbiota affects blood pressure through various mechanisms, including the release of metabolites, cross-kingdom interactions, and immunometabolic pathways. These processes activate blood pressure-regulatory systems, such as the RAAS (151), thereby contributing to the development and progression of hypertension.

As a potential target for hypertension (152), the gastrointestinal tract not only orchestrates food accommodation, turnover, and nutrient absorption but also serves as the body’s most extensive mucosal immune barrier, continuously encountering vast quantities of potentially harmful parasites, bacteria, viruses, fungi, and their derivatives (153). Substantial evidence highlights the potential role of TAS2R activation in innate immunity (154, 155). Activation of TAS2Rs is regarded as a crucial component of gastrointestinal defense mechanisms, regulating the microbiota through immunological pathways (156). Historically, research on TAS2R-microbe interactions has predominantly focused on respiratory diseases (157, 158). Studies indicate that the activation of TAS2R in the airways enhances ciliary beat frequency to expedite microbial clearance (71) and directly initiates antimicrobial responses—such as the release of NO and antimicrobial peptides—to inhibit bacterial proliferation (159). In recent years, research on the interaction between TAS2Rs and microbes has expanded into the gastrointestinal domain. For instance, the level of TAS2R receptor expression is significantly positively correlated with the presence of the probiotic bacterium Akkermansia (39). Additionally, Tas2r105 can indirectly alter the gut microbial community’s structure by influencing the host’s perception of dietary elements (40).

**Figure 7:**
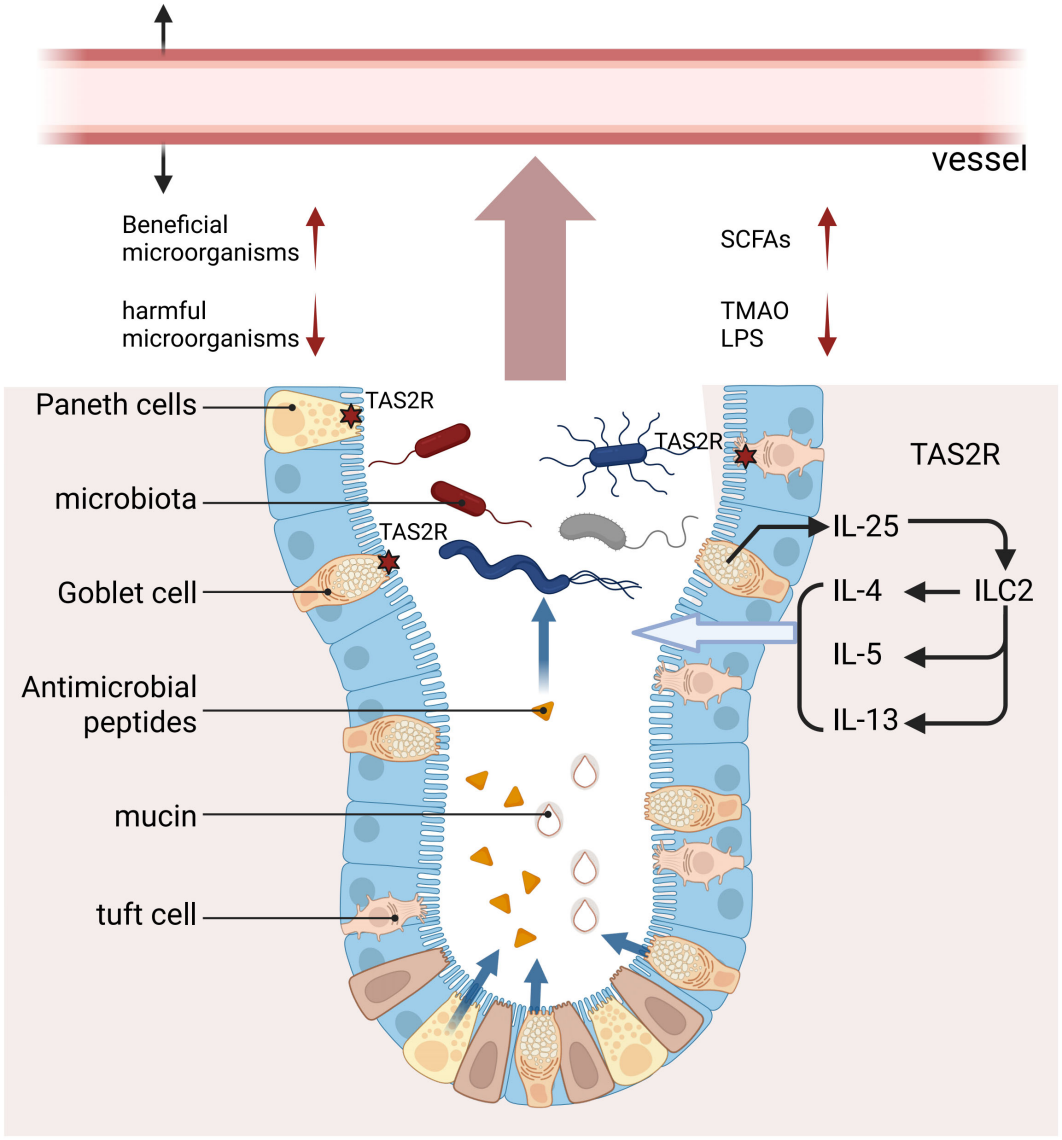
Potential mechanisms of TAS2R Regulating Blood Pressure by Mediating Gut Microbiota: Activation of TAS2R enhances intestinal defense through multiple mechanisms: triggering IL-25-mediated type 2 immunity in tuft cells; stimulating the release of mucins and antimicrobial peptides in goblet and Paneth cells; and promoting intestinal motility to inhibit pathogen colonization. This pathway can be utilized by bitter compounds to exert antihypertensive effects by modulating the gut microbiota and reducing trimethylamine N-oxide (TMAO) levels.

These findings are advancing TAS2R-based strategies for modulating gut microbiota, thereby opening translational avenues for managing gut dysbiosis. TAS2Rs are expressed on immune defense cells within the gastrointestinal tract, including tuft cells, goblet cells, and Paneth cells (75, 160), and they regulate microbiota through multiple pathways to lower blood pressure (**Figure 7**). Firstly, the activation of TAS2Rs in tuft cells triggers type 2 immune responses via IL-25 secretion, thereby enhancing defense against parasites and pathogenic microbes (161). Secondly, the activation of TAS2Rs on goblet and Paneth cells inhibits pathogen growth through the secretion of mucin and antimicrobial peptides (160). Furthermore, the activation of TAS2R promotes intestinal anion secretion, which accelerates the transit of luminal contents, thereby effectively suppressing pathogen colonization (161). Certain interventions have demonstrated antihypertensive effects through the mechanisms previously described. For example, berberine has been shown to activate TAS2R, modulate gut dysbiosis, and subsequently decrease the production of trimethylamine N-oxide (TMAO), thereby contributing to a reduction in blood pressure (162, 163). Similarly, cruciferous vegetables, such as broccoli and cabbage, activate TAS2Rs, which ameliorates gut dysbiosis, alleviates metabolic disorders, and ultimately reduces blood pressure (164).

These findings underscore the potential of TAS2R-based strategies for gut microbiota regulation in the management of hypertension. However, this field remains in its early stages of exploration, facing significant challenges and knowledge gaps. Although research on TAS2R-mediated regulation of the gut microbiome (including bacteria, fungi, and viruses) for blood pressure control shows promising potential with preliminary experimental evidence, the causal relationships remain unclear: whether TAS2R activation or microbiome alterations cause blood pressure changes, or whether blood pressure or dietary factors modulate microbiome composition or TAS2R expression, or whether bidirectional interactions exist. To clarify the complex causal relationships mentioned above, future studies should adopt rigorous experimental designs. For example, employing TAS2R gene knockout models can help distinguish the direct cardiovascular effects resulting from receptor activation from the indirect effects mediated by changes in the gut microbiome. Additionally, comparative experiments between germ-free mice and microbiota-colonized mice are crucial for confirming the causal role of the gut microbiota in this pathway. Furthermore, confounding factors such as dietary patterns, the bioavailability of plant-derived agonists (e.g., polyphenols), and common genetic polymorphisms affecting receptor function (e.g., the TAS2R38 PAV/AVI variants) also significantly complicate the distinction between direct TAS2R-mediated effects and microbiome-dependent pathways. Therefore, future research designs need to integrate standardized dietary control, stratification based on individual genetic backgrounds, and assessments of key metabolite bioavailability to effectively eliminate the influence of these confounding factors, thereby more accurately revealing the independent contributions of TAS2Rs.

#### 4.2.2 Bitter taste receptors and gut microbiota-derived metabolites

Short-chain fatty acids (SCFAs) produced by the gut microbiome play a crucial role in maintaining blood pressure homeostasis, with diminished levels of SCFAs being significantly correlated with the onset of hypertension (165). SCFAs, such as acetate, propionate, and butyrate, exert antihypertensive effects through the activation of G protein-coupled receptors (GPR41, GPR43, and GPR109A), which in turn suppress the renin-angiotensin system and enhance endothelial function (166). Clinical evidence indicates that patients with hypertension exhibit a reduction in gut microbiome diversity and decreased production of SCFAs. Supplementation with SCFAs or probiotic interventions has been shown to effectively reduce blood pressure and improve cardiovascular outcomes (167, 168). TAS2Rs, which function as chemosensory receptors in the gastrointestinal tract, are activated by plant-derived agonists such as epicatechin, resveratrol, and berberine. These agonists have been demonstrated to modulate gut microbiota composition and enhance SCFA synthesis (169–171). SCFAs counteract the pressor effects mediated by angiotensin II through the GPR41/GPR43 signaling pathway (172). In cases of gestational hypertension, intestinal SCFA levels are significantly diminished, and supplementation with butyrate has been shown to exert direct antihypertensive effects (173). Furthermore, SCFAs influence the secretion of gut hormones, including glucagon-like peptide-1 (GLP-1), which plays a role in regulating blood pressure (174, 175). Collectively, these findings underscore the pivotal role of the microbiome-SCFA axis in the regulation of blood pressure, with TAS2Rs acting as crucial regulatory nodes that may translate bitter taste signals into SCFA-mediated protection against hypertension.

#### 4.2.3 Bitter taste receptors enhance gastrointestinal hormone secretion to exert antihypertensive effects

**Figure 8:**
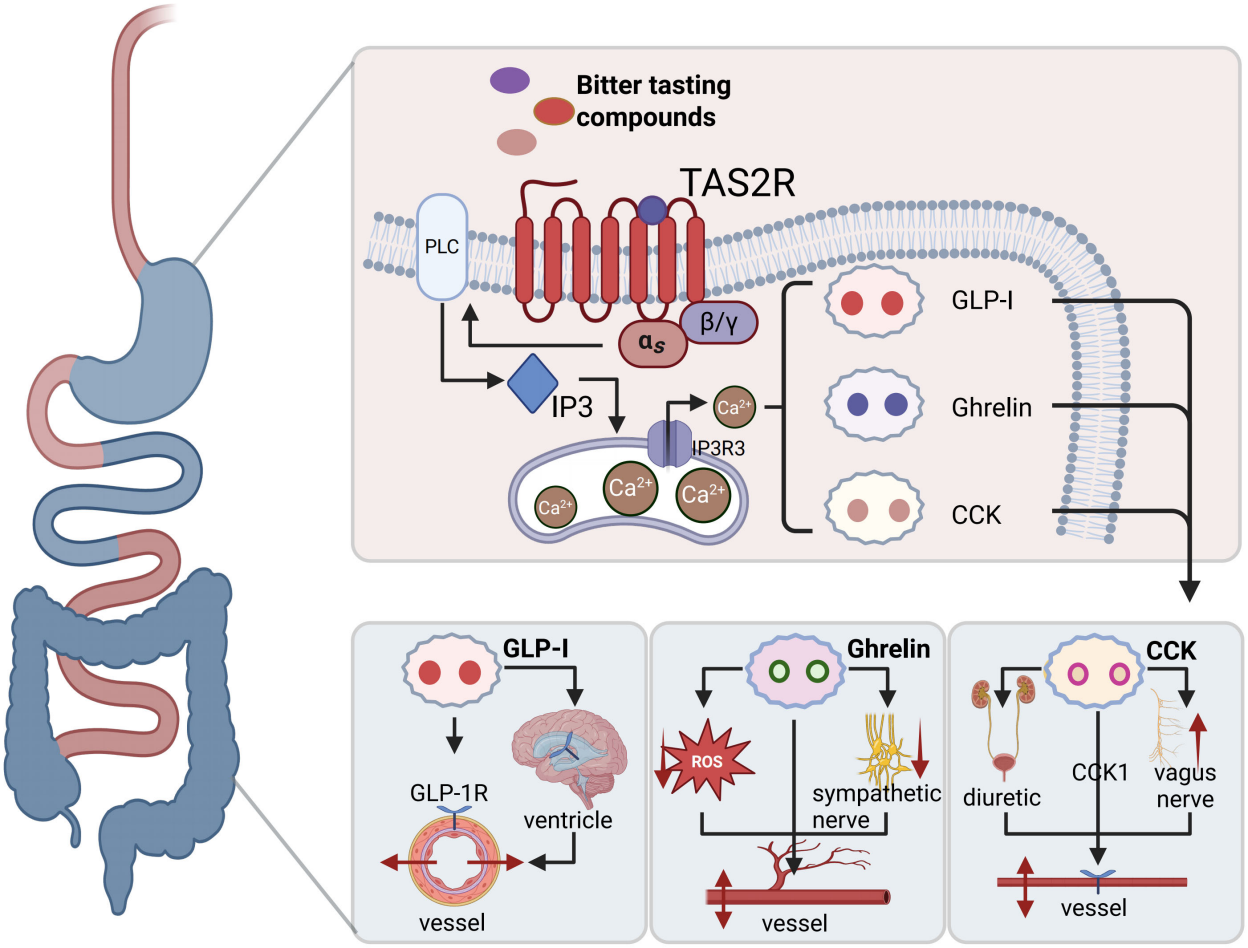
Potential mechanisms of TAS2R Enhancing Blood Pressure Reduction by Regulating Gastrointestinal Hormone Secretion: In gastrointestinal endocrine cells, TAS2R agonists activate TAS2Rs, which leads to the release of increased calcium from calcium channels on the endoplasmic reticulum. This, in turn, stimulates the secretion of GLP-1 and/or CCK and Ghrelin. These hormones can participate in the regulation of blood pressure through multiple pathways.

The activation of the TAS2R signaling pathway significantly induces the secretion of hormones, including GLP-1, ghrelin, and cholecystokinin, which subsequently contribute to the reduction of blood pressure (**Figure 8**).

##### 4.2.3.1 Bitter taste receptors and GLP-1

In recent years, considerable attention has been directed towards the potential role of incretin hormones, specifically GLP-1 and its receptor agonists (GLP-1R), in the management of hypertension (176). GLP-1, an incretin hormone secreted by intestinal L cells, exerts its effects through binding to the GLP-1R and is subsequently inactivated by dipeptidyl peptidase-4 (DPP-4) (177). In addition to its established functions in stimulating insulin secretion and ameliorating insulin resistance, GLP-1 demonstrates a range of pharmacological effects, including anti-inflammatory, neuroprotective, lipid-lowering, weight-reducing, and antihypertensive properties (178).

TAS2Rs are believed to be extensively expressed throughout the body, including within the intestinal tract. Numerous studies have verified the co-expression of TAS2Rs and GLP-1 in intestinal L cells (179, 180). The signaling pathway of bitter taste receptors constitutes a crucial mechanism for the regulation of GLP-1 secretion (181). Agonists of TAS2R, such as denatonium benzoate that targets TAS2R4, TAS2R43, and TAS2R46, and ofloxacin, a specific agonist of TAS2R9, both effectively enhance GLP-1 secretion from intestinal enteroendocrine L cells (182, 183). Specifically, the binding of TAS2Rs agonists to gastrointestinal TAS2R induces conformational changes in the receptors, which trigger downstream signaling cascades and membrane depolarization, ultimately stimulating intestinal L cells to secrete GLP-1 (184). GLP-1 mediates its extensive pharmacological effects by binding to the GLP-1R. It has been established that GLP-1R is expressed not only in vascular endothelial and smooth muscle cells (185, 186) but is also widely distributed in the circumventricular organs of rats, including the area postrema, subfornical organ, median eminence, and the vascular organ of the lamina terminalis, as well as in the arcuate nucleus and nucleus tractus solitarius. These regions constitute central core areas involved in the regulation of blood pressure (187). Research suggests that diminished expression of the GLP-1 receptor in cardiovascular metabolic conditions, such as hypertension, is linked to sympathetic overactivity (188).

Consequently, the modulation of intestinal bitter taste signaling pathways to augment endogenous GLP-1 secretion has the potential to reduce blood pressure through various mechanisms, including direct vasodilation and influences on central and peripheral blood pressure regulatory receptors (**Figure 7**). This process may constitute a critical element of the gut-brain-vascular axis interactions involved in blood pressure regulation, offering novel perspectives for the development of future GLP-1-based antihypertensive therapies.

##### 4.2.3.2 Bitter taste receptors and ghrelin

Ghrelin, predominantly secreted by gastric cells, serves as a peptide hormone that regulates gastric motility, mediates hunger signals in the brain, promotes food intake, and stimulates the release of growth hormone (189). Recent studies, however, have identified additional roles of ghrelin within the cardiovascular system, including its anti-inflammatory properties, sympathetic inhibition, and vasodilatory effects, which are crucial in the regulation of blood pressure and the development of hypertension (190, 191). Patients with hypertension consistently exhibit reduced circulating levels of ghrelin, which are inversely correlated with blood pressure (192, 193). Increasing circulating ghrelin concentrations has been shown to reduce blood pressure (194, 195), potentially through the activation of AMPK signaling, the inhibition of oxidative stress, and the enhancement of vascular endothelial function (196). Furthermore, ghrelin offers dual advantages for blood pressure regulation and vascular health by suppressing vascular inflammation (197) and inhibiting the proliferation of vascular smooth muscle cells (**Figure 7**) (198). Consequently, the promotion of ghrelin secretion could constitute a novel approach to managing hypertension. Recent research has demonstrated that TAS2Rs are present in gastric cells, where agonists of these receptors stimulate ghrelin secretion from gastric fundus cells (199), predominantly through the α-gustducin-coupled signaling pathway (200). These findings imply that targeting intestinal TAS2Rs to augment ghrelin secretion may represent an innovative therapeutic strategy for adjunctive blood pressure reduction.

##### 4.2.3.3 Bitter taste receptors and cholecystokinin

Cholecystokinin (CCK), originally identified for its role in stimulating pancreatic secretion (201), was subsequently recognized for its ability to induce gallbladder contraction, leading to its designation as CCK in 1928 (202). CCK encompasses several subtypes, including CCK4, CCK5, CCK6, CCK8, CCK12, CCK18, CCK39, and CCK58 peptides (203). Secreted by the duodenum, CCK functions as both a gastrointestinal hormone and a neuropeptide, playing a pivotal role in the regulation of satiety and appetite suppression (204). Furthermore, CCK confers multiple benefits in the regulation of blood pressure. Firstly, increased circulating levels of CCK bind to CCK1 receptors, promoting vasodilation and consequently reducing blood pressure in rats (205, 206). Secondly, in hypertensive rat models, CCK enhances renal blood flow, facilitating diuresis and maintaining sodium homeostasis (207–209). Thirdly, CCK acts on vagal afferent nerves to reflexively inhibit renal and visceral sympathetic nerves, thereby inducing vasodilation (210). This suggests the potential therapeutic value of CCK in the management of hypertension (**Figure 7**). TAS2Rs are extensively expressed in duodenal epithelial cells, and their activation facilitates the secretion of CCK (211–213). Research indicates that CCK plays a dual role by modulating gastric emptying, thereby contributing to gut defense mechanisms, and by mediating vasodilation (214). Consequently, TAS2R may exert antihypertensive effects through the secretion of CCK.

Different subtypes of TAS2R play distinct roles in modulating the secretion of various gastrointestinal hormones. For instance, the activation of TAS2R5 is associated with the release of GLP-1 and CCK, TAS2R10 activation is linked to increased ghrelin secretion, and TAS2R14 activation promotes GLP-1 secretion. Considering the complexity and multifactorial nature of the effects arising from the simultaneous activation of multiple receptors, identifying TAS2R subtypes that synergistically enhance the secretion of multiple gastrointestinal hormones could represent a valuable avenue for future research.

## 5 Bitter taste receptor agonists with potential for hypertension treatment

Research has demonstrated that various agonists of TAS2R possess considerable antihypertensive potential (**Table 3**), thereby offering novel strategies for the treatment of hypertension. In particular, clinical investigations have highlighted the efficacy of quercetin, a TAS2R14 agonist, in significantly reducing blood pressure, as evidenced by systematic reviews and meta-analyses encompassing 17 clinical trials (225, 235, 236, 242). Similarly, proanthocyanidins, acting as a TAS2R5 agonist, have been confirmed to exert antihypertensive effects according to systematic reviews and meta-analyses of 19 clinical trials (225, 226, 242). Furthermore, systematic reviews and meta-analyses of 27 randomized controlled clinical trials suggest that berberine, a TAS2R38 agonist, is more effective in lowering blood pressure compared to lifestyle interventions alone or placebo (242, 243). Additionally, meta-analytic evidence regarding oleuropein, a TAS2R8 agonist, indicates a positive correlation between oral intake and improved hypertension outcomes, thereby reinforcing the clinical significance of activating TAS2R (227, 242). In the context of treatment strategies for specific populations, the combination of low-dose dextromethorphan (an agonist of TAS2R1 and TAS2R10) with amlodipine has been shown to yield significant synergistic antihypertensive effects in patients with hypertension and endothelial dysfunction (219, 225). Additionally, colchicine (an agonist of TAS2R4, TAS2R39, and TAS2R46) has been validated to restore vasodilatory function in hypertensive patients through multiple mechanisms (222, 225). Moreover, there is a negative correlation between serum thiamine levels and both the prevalence of hypertension and systolic blood pressure in middle-aged and elderly women, thereby providing epidemiological support for the inclusion of thiamine (a TAS2R1 agonist) supplementation in hypertension prevention strategies (218, 225).

**Table 3:** Bitter taste receptor agonists with therapeutic potential for hypertension.

Bitter taste receptors agonists	Bitter taste receptors	Association with hypertension	Refs
Amygdalin	1,4,30,39, 43,46,50	Amygdalin demonstrates inhibitory activity against angiotensin-converting enzyme I (ACE-I), an enzyme implicated in hypertension pathogenesis.	(215)
Parthenolide	1,4,8,10,14, 31,46	Parthenolide exhibits inhibition of arterial vascular proliferation and migration.Parthenolide attenuates vascular inflammation.	(216, 217)
Thiamine	1	Serum thiamine levels are inversely correlated with hypertension prevalence and systolic blood pressure in middle-aged and elderly women, supporting thiamine’s potential role in hypertension prevention strategies.	(218)
Dextromethorphan	1,10	The combination of low-dose dextromethorphan (DXM) and amlodipine (AM) confers significant antihypertensive benefits in patients with hypertension and impaired endothelial function.	(219)
Chloroquine	3,7,10,39	Chloroquine administration (40 mg/kg/day, intraperitoneal) significantly reduced blood pressure in spontaneously hypertensive rats (SHR).	(220, 221)
Colchicine	4,39,46	Colchicine therapy restores vascular vasodilation in patients with hypertension.	(222)
Epicatechin	4,5,39	In spontaneously hypertensive rats (SHR), high-dose epicatechin administration significantly attenuated blood pressure, with this antihypertensive effect persisting for two weeks following treatment discontinuation.	(223, 224)
proanthocyanidins	5	A systematic review and meta-analysis of 19 randomized controlled trials (RCTs) indicates that proanthocyanidins (grape seed extract) exert blood pressure-lowering effects.	(225, 226)
Oleuropein	8	Meta-analytical evidence demonstrates a positive association between oral oleuropein intake and improved hypertension outcomes.	(227)
Cucurbitacin B	10,14	Preclinical studies indicate that cucurbitacin B lowers systolic blood pressure through induction of vasodilation.	(80)
Cucurbitacin E	10	Long-term dietary supplementation with cucurbitacin E ameliorates high-salt-induced cardiovascular dysfunction and angiotensin II-induced hypertension.	(228)
Liensinine	10,46	Liensinine exerts antihypertensive effects against angiotensin II (Ang II)-induced hypertension.	(229)
Naringenin	14	Multiple preclinical studies demonstrate the antihypertensive properties of naringenin.	(230)
Baicalein	14,39	Preclinical evidence consistently supports the antihypertensive efficacy of baicalein.	(231)
Limonin	14,38	Several studies highlight the metabolic benefits and antihypertensive potential of limonin.	(232–234)
Quercetin	14	A meta-analysis of 17 clinical trials demonstrated that quercetin significantly lowers blood pressure, warranting its consideration as an adjunctive therapy for hypertensive patients.	(235, 236)
Kaempferol	14,39	The vasodilatory effect of kaempferol is well-established; however, direct antihypertensive efficacy has not yet been demonstrated in clinical studies.	(237, 238)
Luteolin	14	Preclinical investigations consistently report antihypertensive effects of luteolin.	(239, 240)
Andrographolide	30,46,50	Andrographolide not only reduces blood pressure but also confers cardiovascular protective effects.	(241)
Aloin	31,43	Preliminary evidence suggests that aloe-emodin possesses antihypertensive properties.	(230)
Resveratrol	14	Resveratrol effectively lowers blood pressure through anti-inflammatory effects and inhibition of vascular smooth muscle proliferation.	(237)

In animal models and preclinical studies, a growing body of evidence indicates that various bitter compounds exhibit antihypertensive effects through the activation of TAS2R. In models of spontaneously hypertensive rats, administration of high-dose epigallocatechin, an agonist of TAS2R4, TAS2R5, and TAS2R39, significantly reduces blood pressure, with these antihypertensive effects persisting for two weeks following cessation of treatment (223, 224, 242). Similarly, intraperitoneal injection of chloroquine, an agonist of TAS2R3, TAS2R7, TAS2R10, and TAS2R39, at a dosage of 40 mg/kg/day, also significantly lowers blood pressure in spontaneously hypertensive rats (220, 221, 225). Furthermore, cucurbitacin B, an agonist of TAS2R10 and TAS2R14, and cucurbitacin E, an agonist of TAS2R10, exhibit blood pressure-lowering effects via activation of the TAS2R pathway. Cucurbitacin B induces vasodilation, thereby reducing systolic blood pressure, while cucurbitacin E ameliorates cardiovascular dysfunction induced by a high-salt diet (80, 228). Additionally, neferine, an agonist of TAS2R10 and TAS2R46, has been shown to exert antihypertensive effects against angiotensin II-induced hypertension (229, 242). Among flavonoids, baicalein (an agonist of TAS2R14 and TAS2R39), luteolin (an agonist of TAS2R14), resveratrol (an agonist of TAS2R14), and kaempferol (an agonist of TAS2R14 and TAS2R39) have demonstrated antihypertensive properties mediated by TAS2Rs in preclinical studies. Notably, the vasodilatory effects of kaempferol have been substantiated in multiple investigations (231, 237–240, 242, 244). Furthermore, andrographolide, which acts as an agonist for TAS2R30, TAS2R46, and TAS2R50, not only reduces blood pressure through the activation of TAS2R but also exhibits cardiovascular protective effects (241, 242). Preliminary studies on limonin (an agonist of TAS2R14 and TAS2R38), naringenin (TAS2R14 agonist), and aloin (TAS2R31, TAS2R43 agonist) also suggest their potential antihypertensive effects via TAS2R pathways (230, 232–234, 242). Additionally, amygdalin (an agonist of TAS2R1, TAS2R4, TAS2R30, TAS2R39, TAS2R43, TAS2R46, and TAS2R50) and parthenolide (an agonist of TAS2R1, TAS2R4, TAS2R8, TAS2R10, TAS2R14, TAS2R31, and TAS2R46), as multi-receptor agonists, further expand the repertoire of compounds available for targeting hypertension through TAS2Rs (215–217, 225, 245).

Nonetheless, the majority of these bitter compounds exhibit generally low oral bioavailability (246), a characteristic that appears to contradict their extensive pharmacological effects observed *in vivo*. This discrepancy strongly implies that signaling pathways mediated by gut TAS2Rs may play a pivotal role in facilitating antihypertensive effects, rather than relying exclusively on conventional multi-target mechanisms (246–248). Furthermore, to overcome the challenge of limited bioavailability of bitter compounds, researchers are devising innovative strategies involving structural modification and advanced drug delivery systems (249–251). In conclusion, related research not only establishes a theoretical foundation for the development of TAS2R agonists as novel antihypertensive drugs but also identifies lead compounds with significant translational potential.

## 6 Unmet needs and future directions

TAS2Rs serve as crucial regulators within the gut-vascular axis and demonstrate significant potential as antihypertensive therapeutic agents. As previously discussed, upon activation by bitter agonists at gastrointestinal and vascular interfaces, these receptors mediate various blood pressure-lowering effects through the regulation of vasodilation, metabolic homeostasis, and immunomodulatory pathways. This novel framework has substantially enhanced our understanding of the roles that TAS2Rs play in hypertension. Furthermore, it presents an opportunity to target TAS2Rs as adjunctive therapy for hypertension. Nonetheless, further research is required to facilitate their translation and application in clinical settings.

(1) Development of high-selectivity agonists, antagonists, and related antibodies.

Owing to the absence of specific agonists and antagonists targeting TAS2R, the majority of studies are limited to examining the downstream signaling molecules within the TAS2R pathways. As a result, the evidentiary link connecting the multifunctional effects of bitter compounds to TAS2R is incomplete and necessitates further elucidation through genetic knockout methodologies. Moreover, given the numerous TAS2R subtypes, it remains uncertain which specific subtypes are activated by bitter compounds in the regulation of vasodilation, metabolic homeostasis, and immunomodulation. The binding interactions between TAS2R and bitter compounds also remain unidentified. Future research should prioritize the development of TAS2R-specific pharmacological tools and related antibodies as essential objectives for advancing the study of TAS2R expression and function.

Currently, the development of selective TAS2R antagonists is still in its early stages. In complex physiological systems where multiple receptor subtypes are co-expressed, highly selective antagonists hold irreplaceable value for deciphering TAS2R-mediated physiological responses. However, the number of existing TAS2R antagonists is limited, and their specificity is generally insufficient. Taking the TAS2R14 subtype as an example, only three antagonists are currently known, while the number of agonists for the same receptor exceeds 150, highlighting the severe lag in antagonist development (252). Identified synthetic antagonists, such as GIV3727, similar to agonists, generally lack specificity. For instance, GIV3727 not only blocks its initial targets TAS2R31 and TAS2R43 but also inhibits several other TAS2R subtypes (253). Similarly, natural compounds such as 4’-fluoro-6-methoxyflavanone and 6-methoxyflavanone have also been shown to inhibit multiple TAS2R subtypes (254). Secondly, certain molecules (e.g., 3β-hydroxydihydrocostunolide and 3β-hydroxypelenolide) even exhibit functional duality—acting as antagonists for one subtype while potentially serving as partial agonists for others—further complicating the development of highly selective antagonists (255). Furthermore, drugs such as probenecid achieve non-competitive inhibition of bitter taste receptors (e.g., TAS2R16, TAS2R38, and TAS2R43) by targeting their intracellular loop regions through allosteric modulation. This mechanism significantly heightens both the design complexity and technical barriers associated with developing highly selective antagonists (256).

These limitations stem from multiple challenges: first, the long-standing lack of experimental three-dimensional structural data has severely hindered structure-based rational drug design (252); second, the inherent broad ligand recognition properties of the receptors and the high conservation of binding pockets make achieving subtype-specific inhibition extremely difficult (257); furthermore, the complexity of functional expression and screening technologies for TAS2Rs in heterologous systems also constrains high-throughput antagonist discovery (257). Notably, recent breakthroughs in resolving cryo-electron microscopy structures of TAS2R46 and TAS2R14, among others, have provided new opportunities for rational design (45, 258). Future strategies should focus on iterative hybrid methodologies—integrating experimental screening (such as exploring new uses for known drug libraries), computational optimization (such as virtual screening and molecular docking) to elucidate key activation and inhibition mechanisms, and leveraging algorithms like BitterMatch to predict ligand selectivity, while incorporating new sensing technologies (such as bioelectronic tongues) to enhance the accuracy and throughput of functional validation (252, 257, 259). These advances are not only crucial for elucidating the pathophysiological functions of extraoral TAS2Rs in tissues such as blood vessels, the gastrointestinal tract, and macrophages but also open new pathways for their potential therapeutic applications. There is an urgent need for interdisciplinary systematic research to address these gaps in the future.

(2) Exploration of bitter taste receptor expression in health versus hypertension states.

Research on the differential expression of TAS2R between healthy individuals and those with hypertension is limited. Major challenges in human tissue studies include inherently low TAS2R mRNA abundance in extraoral tissues and the absence of rigorously validated antibodies for protein detection. Consequently, it remains unknown whether TAS2R expression within the gut-vascular axis is unchanged or significantly reduced in hypertension. To address these limitations, emerging methodologies must be prioritized. Single-cell RNA sequencing (260) and spatial transcriptomics (261) can provide high-resolution cellular mapping of TAS2R expression patterns. Human organoid models of vascular or gastrointestinal tissues integrated with microfluidic organ-on-a-chip platforms (262) offer physiologically relevant systems to assess receptor dynamics under hypertensive conditions. Future studies must define disease-specific TAS2R expression profiles using these advanced methodologies while accounting for species-specific receptor repertoire differences. This knowledge is fundamental to establishing TAS2R dysfunction in hypertension pathogenesis and identifying patients amenable to TAS2R-targeted adjunctive therapy.

(3) Advancement of studies combining bitter compounds with clinically used antihypertensive drugs.

Previous studies have shown that the combination of bitter compounds, low-dose dextromethorphan, and antihypertensive medications results in significant synergistic effects in reducing blood pressure among hypertensive patients (219). Bitter compounds offer a range of benefits, including glucose-lowering, lipid-lowering, blood pressure reduction, and vascular protection (263). Future research endeavors should focus on advancing both basic and clinical investigations into the combination of bitter compounds with antihypertensive drugs. This approach may represent a promising pathway for expediting the clinical application of TAS2R agonists as adjunctive therapies for hypertension management.

(4) Advancement of endogenous bitter taste receptor discovery.

The extensive expression of TAS2Rs in non-gustatory tissues strongly indicates the presence and physiological roles of endogenous agonists, specifically ligands synthesized by the body (264). Recent studies have intriguingly identified cholesterol (265, 266), advanced glycation end products (116), and bile acids (267) as endogenous bitter agonists. Nonetheless, there remains a significant paucity of information concerning the identities, origins, regulatory mechanisms, and precise functions of additional endogenous agonists within non-gustatory systems. This represents a substantial gap in knowledge that necessitates urgent advancements in research within this domain.

(5) Exploration of synergistic blood pressure regulation strategies with TRPV1.

The concurrent utilization of bitter and pungent compounds, such as chili peppers, has a longstanding tradition in traditional Chinese medicine. The active components of pungent compounds are detected by the TRPV1 receptor (268), whereas bitter compounds primarily interact with TAS2R. Contemporary research indicates a significant integration of bitter and nociceptive signals within higher neural centers, with neurons in the parabrachial nucleus (PBN) functioning as critical hubs (269). Optogenetic activation of TRPV1-positive fibers in the spinal trigeminal tract markedly enhances the activity of PBN neurons responsive to bitter stimuli, thereby confirming the modulation of bitter perception by nociceptive signals (270). Conversely, in pulmonary nociceptors, agonists of TAS2R, such as chloroquine, substantially augment TRPV1-mediated currents via the phospholipase C (PLC) and protein kinase C (PKC) signaling pathways (271). This suggests the presence of bidirectional regulatory mechanisms between TAS2R and TRPV1 channels. Moreover, research has demonstrated that the concurrent activation of bitter taste receptors and TRPV1 results in a synergistic therapeutic enhancement across various disease treatments (272–274). Considering the extensively documented significance of TRPV1 in hypertension (275–277), alongside the comprehensive analysis of the pivotal roles of TAS2R in hypertension within this review, the simultaneous regulation of TAS2R and TRPV1 emerges as a promising avenue for further investigation in the context of future hypertension therapies.

## 7 Conclusion

Hypertension is a multifaceted syndrome characterized by the involvement of various factors and organ systems, with pathophysiological mechanisms that include interactive effects across neural, humoral, metabolic, and immune systems. This review systematically integrates multidimensional evidence regarding the role of TAS2R within the gut-vascular axis in the regulation of blood pressure, highlighting the potential significance of this previously underexplored molecular system in the maintenance of blood pressure homeostasis. Current research indicates that TAS2R are not only extensively expressed at cardiovascular interfaces but also establish indirect regulatory networks at gastrointestinal interfaces, thereby constituting the “gut-vascular axis” that links gut microbiota with vascular function. Current evidence objectively suggests that TAS2R are involved in blood pressure regulation through various mechanisms. This multi-organ and multi-pathway regulatory capability positions TAS2R as potential integrative nodes linking immune, metabolic, and neural networks, thereby offering novel insights into this intricate pathological process. Nonetheless, it is important to acknowledge that research into the mechanistic roles and clinical significance of TAS2R in human hypertension is still in its nascent stages. The majority of the evidence is derived from *in vitro* studies and animal models, with human data being relatively scarce and largely observational. In conclusion, investigating TAS2Rs within the gut-vascular axis offers new perspectives on the complex pathophysiological mechanisms underlying hypertension, and targeting TAS2R may emerge as a promising strategy for comprehensive hypertension management.
